# COVID-19 psychological impact in 3109 healthcare workers in Spain: The PSIMCOV group

**DOI:** 10.1017/S0033291720001671

**Published:** 2020-05-14

**Authors:** Carolina S. Romero, Carlos Delgado, Juan Catalá, Carolina Ferrer, Carlos Errando, Adina Iftimi, Ana Benito, Jose de Andrés, Maria Otero, The PSIMCOV group*

**Affiliations:** 1 Department of Anaesthesia, Valencia University General Hospital, Valencia, Spain; 2 Department of Statistics, O.R. Universitat de València, E- 46100-Burjassot, Spain; 3 Department of Psychiatry, Valencia University General Hospital, Valencia, Spain; 4 Department of the Multidisciplinary Pain Management, Valencia University Medical School, Valencia University General Hospital, Valencia, Spain

**Keywords:** Covid-19, healthcare workers, mental health, pandemic, psychological, SARS-CoV-2

## Abstract

**Background:**

The current coronavirus disease (COVID-19) has a great impact worldwide. Healthcare workers play an essential role and are one of the most exposed groups. Information about the psychosocial impact on healthcare workers is limited.

**Methods:**

3109 healthcare workers completed a national, internet-based, cross-sectional 45-item survey between 9 and 19 April 2020. The objective is to assess the psychological impact of the COVID-19 pandemic in Spanish healthcare workers. A Psychological Stress and Adaptation at work Score (PSAS) was defined combining four modified versions of validated psychological assessment tests (A) *Healthcare Stressful Test*, (B) *Coping Strategies Inventory*, (C) *Font-Roja Questionnaire* and (D) *Trait Meta-Mood Scale*.

**Results:**

The highest psychosocial impact was perceived in Respiratory Medicine, the mean (S.D.) PSAS was 48.3 (13.6) and Geriatrics 47.6 (16.4). Higher distress levels were found in the geographical areas with the highest incidence of COVID-19 (>245.5 cases per 100 000 people), PSAS 46.8 (15.2); *p* < 0.001. The least stress respondents were asymptomatic workers PSAS, 41.3 (15.4); *p* < 0.001, as well as those above 60 years old, PSAS, 37.6 (16); *p* < 0.001. Workers who needed psychological therapy and did not receive it, were more stressed PSAS 52.5 (13.6) than those who did not need it PSAS 39.7 (13.9); *p* < 0.001.

**Conclusions:**

The psychological impact in healthcare workers in Spain during COVID-19 emergency has been studied. The stress perceived is parallel to the number of cases per 100 000 people. Psychotherapy could have a major role to mitigate the experimented stress level.

Many efforts in the clinical field of the coronavirus disease (COVID-19) are being made. However, mental health is also at stake during this outbreak. Psychological distress is already being detected among the healthcare professionals in Asia (Casas, Repullo, & Lorenzo, 2002; Xiao, Zhang, Kong, Li, & Yang, 2020; Yuan et al., 2020). Information on the psychological impact of healthcare workers is still limited in European countries. Knowledge of this impact is crucial to establish a Mental Health Crisis Response (Pfefferbaum & North 2020). This study describes the psychological stress experimented by the healthcare workers involved in the COVID-19 outbreak in Spain.

This national, internet-based, cross-sectional survey was performed by the Research Institute of the University General Hospital of Valencia, which was the coordinating center for the Psychological Impact of Coronavirus (PSIMCOV) network. For the stress and psychological impact evaluation, four modified versions of validated tests (Appendix 1), were considered to match a context within the extreme shortage of time; (A) *Healthcare Stressful Test* for identifying stressing factors at work (Cano, Rodríguez, & García, 2007; Carver, Scheier, & Weintraub, 1989), (B) *Coping Strategies Inventory* for assessing problem solving, self-criticism, emotional expression, willing thoughts, social support, problem avoidance and social support spheres (Aranaz, Mira & Font-Roja Questionnaire, 1988; Salovey, Mayer, Goldman, Turvey, & Palfai, 1995; Tobin, Holroyd, Reynolds, & Kigal, 1989), (C) *Font-Roja Questionnaire* for assessing satisfaction, pressure, relationships, relaxation, adequacy, control and task variety at work (Fernández-Berrocal & Extremera, 2006) and (D) *Trait Meta-Mood Scale* for assessing interpersonal aspects of emotional intelligence (Haynes & Lench, 2003; Johnston & Murray, 2003). Every assessed area was represented by at least one question. We defined the *Psychological Stress and Adaptation at work Score* (*PSAS*) as a combined measure of the scores obtained in each of the four tests described.

Data were analyzed using the statistical software R (Core Team, 2013). The *p* values in the tables were calculated with one-way analysis of variance (ANOVA) comparing the mean of *PSAS*. Variables *region* and *psychotherapy* were studied with ANOVA analysis and a Tukey's test for multiple comparisons of means. For the variable *Children <12 years old, elderly or handicapped at home*, we carried out a *t* test.

A total of 3109 surveys were analyzed from 9 to 19 April 2020, the most epidemiologically stressful stage of the emergency. Table 1 shows demographics and the main characteristics of the participants of the study. Table 2 shows the global psychological impact results measured by *PSAS*. *Age* and the stress perceived, are inversely correlated (*p* < 0.0001) as seen in a linear regression model reflected in Fig. 1. For analytical purposes, the Spanish geography was divided into five areas based on cumulative incidences defined by the National Health Authority. Healthcare workers in the areas with a higher number of cases (Group V), showed a higher degree of stress globally and in each separated test (*p* < 0.0001) with a mean (s.d.), PSAS 46.8 (15.2).

**Fig. 1.**
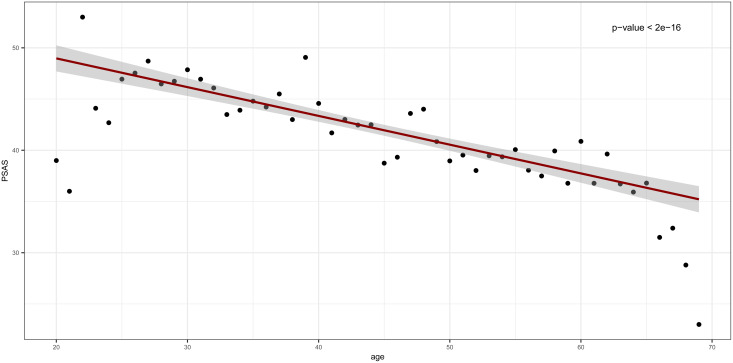
Linear regression between the variables Age and PSAS.

**Table 1.** Characteristics of the respondents

Characteristic	No.	%	Total
			*n* = 3109
Age mean (s.d.) – year			45.14 (6.48)
Age category – no. (%)			
<20 years	2	0.1	2 (0.1%)
20–29 years	350	11.3	350 (11.3%)
30–39 years	737	23.7	737 (23.7%)
40–49 years	895	28.8	895 (28.8%)
50–59 years	781	25.1	781 (25.1%)
60–69 years	334	10.7	334 (10.7%)
⩾70 years	8	0.3	8 (0.3%)
Area – no. (%)*			
Group I	105	3.4	105 (3.4%)
Group II	2089	67.2	2089 (67.2%)
Group III	71	2.3	71 (2.3%)
Group IV	369	11.9	369 (11.9%)
Group V	475	15.3	475 (15.3%)
Category – no. (%)			
Medical staff	1761	56.6	1761 (56.6%)
Nursing staff	825	26.5	825 (26.5%)
Nurse assistants	238	7.7	238 (7.7%)
Ancillary staff	34	1.1	34 (1.1%)
Administrative staff	48	1.5	48 (1.5%)
Laboratory technicians	24	0.8	24 (0.8%)
Researcher and faculty members	27	0.9	27 (0.9%)
Pharmaceutical representatives	21	0.7	21 (0.7%)
Management staff	12	0.4	12 (0.4%)
Hospital pharmacists	69	2.2	69 (2.2%)
Others	50	1.6	50 (1.6%)
Medical specialty – no. (%)			
Allergy	30	1	30 (1%)
Clinical analysis	17	0.6	17 (0.6%)
Pathology	320	10.5	320 (10.5%)
Anesthesiology and Critical Care	766	25.2	766 (25.2%)
Cardiology	52	1.7	52 (1.7%)
Cardiac surgery	48	1.6	48 (1.6%)
General surgery	109	3.6	109 (3.6%)
Orthopedic and trauma medicine	75	2.5	75 (2.5%)
Vascular surgery	13	0.4	13 (0.4%)
Thoracic surgery	17	0.6	17 (0.6%)
Dermatology	21	0.7	21 (0.7%)
Hospital Pharmacy	35	1.2	35 (1.2%)
Gastroenterology	29	1	29 (1%)
Obstetrics and Gynecology	104	3.4	104 (3.4%)
Geriatrics	25	0.8	25 (0.8%)
Hematology	29	1	29 (1%)
Home care doctors	20	0.7	20 (0.7%)
Infectious diseases	14	0.5	14 (0.5%)
Emergency Medicine	135	4.4	135 (4.4%)
Physical medicine and Rehabilitation	31	1	31 (1%)
Intensivists and Critical Care	157	5.2	157 (5.2%)
Internal Medicine	105	3.5	105 (3.5%)
Nephrology	12	0.4	12 (0.4%)
Neurosurgery	29	1	29 (1%)
Neurology	30	1	30 (1%)
Ophthalmology	33	1.1	33 (1.1%)
Medical Oncology	13	0.4	13 (0.4%)
Otorhinolaryngology	31	1	31 (1%)
Others	332	10.9	332 (10.9%)
Pediatrics	131	4.3	131 (4.3%)
Psychiatry	38	1.2	38 (1.2%)
Radiology	72	2.4	72 (2.4%)
Respiratory Medicine	51	1.7	51 (1.7%)
Urology	19	0.6	19 (0.6%)
Workplace			
Primary hospital	159	7	159(7%)
Secondary hospital	193	8.5	193 (8.5%)
Tertiary hospital	1185	52.5	1185 (52.5%)
General practitioners in medical centers	293	13	293 (13%)
Ambulance services	429	19	429 (19%)
Seniority			
0–1 year	379	12.2	379 (12.2%)
1–3 years	270	8.7	270 (8.7%)
3–5 years	434	14	434 (14%)
5–10 years	302	9.7	302 (9.7%)
10–20 years	872	28	872 (28%)
More than 20 years	849	27.3	849 (27.3%)

*Group I: 19.7–33 cases per 100 000 people.

Group II: 34–70.8 cases per 100 000 people.

Group III: 70.9–117.9 cases per 100 000 people.

Group IV: 118–245.8 cases per 100 000 people.

Group V: 245.9–351.3 cases per 100 000 people.

**Table 2.** Psychological impact on the healthcare workers

	Test A	Test B	Test C	Test D	PSAS	*p* value**
	Healthcare stressful factors test	Coping strategies inventory	Font-Roja Questionnaire	Trait meta-mood scale		
Subgroup	Mean (s.d.)					
Age category						
20–29 years	5.9 (2.3)	18.5 (6.4)	15.5 (6.3)	6.8 (3.5)	46.7 (14.8)	<0.001
30–39 years	5.7 (2.5)	17.1 (6.6)	16.1 (6.5)	6.6 (3.8)	45.5 (15.6)	
40–49 years	5.3 (2.5)	16 (6.6)	14.9 (6.5)	5.9 (3.6)	42.1 (15.1)	
50–59 years	5.1 (2.3)	14.9 (6.4)	13.2 (6.4)	5.7 (3.6)	38.8 (14.5)	
60–69 years	5.1 (2.5)	13.7 (6.6)	13.1 (6.6)	5.7 (3.8)	37.6 (16)	
Region						<0.001
Group I	4.8 (2.3)	15.4 (6.9)	13.2 (6.8)	5.7 (3.4)	39.1 (15)	
Group II	5.3 (2.4)	15.7 (6.7)	14 (6.4)	6.1 (3.7)	41 (15.2)	
Group III	5.5 (2.7)	16 (6.7)	15.1 (6.3)	5.1 (3.4)	41.8 (14.9)	
Group IV	5.6 (2.4)	15.8 (6.7)	15.6 (6.6)	6.1 (3.7)	43.1 (16.2)	
Group V	5.9 (2.5)	17.7 (6.3)	16.5 (6.7)	6.8 (3.8)	46.8 (15.2)	
Category						
Medical staff	5.4 (2.5)	15.7 (6.7)	15.3 (6.6)	5.9 (3.6)	42.3 (15.8)	
Nursing staff	5.7 (2.4)	16.8 (6.4)	14 (6.2)	6.5 (3.7)	43 (14.7)	
Nurse assistants	5.1 (2.4)	15.9 (6.9)	13.1 (6.8)	6.5 (4.1)	40.6 (16)	
Ancillary staff	5.2 (2.6)	15 (7.1)	13.4 (7.1)	6.4 (4.1)	40 (16.6)	
Management staff	4.8 (2.7)	12.4 (7.9)	14.8 (8.6)	4.2 (3)	36.2 (19.7)	
Hospital Pharmacists	5.1 (2.5)	16.6 (6.5)	14.1 (6.1)	6.6 (4)	42.3 (14.4)	
Medical speciality						
Allergy	5.1 (2.5)	16.8 (6.7)	15.6 (6.8)	6.6 (4.1)	44 (15.3)	
Clinical analysis	4.8 (2.5)	16.2 (7.4)	13.7 (7.3)	6.9 (3)	41.6 (16)	
Anesthesiology and Critical Care	5.7 (2.4)	15.6 (6.5)	15.1 (6.6)	5.8 (3.6)	42.3 (15.6)	
Cardiology	5.7 (2.7)	14.9 (6.5)	14.4 (4.9)	6.9 (3.6)	41.9 (13.1)	
Cardiac surgery	5.1 (2.1)	14.6 (5.9)	11.6 (6.1)	5.4 (3)	36.7 (13.8)	
General surgery	5.2 (2.3)	15.6 (6)	14.5 (6.3)	5.3 (3.8)	40.6 (14.3)	
Hospital Pharmacy	4.7 (1.9)	18.9 (6.8)	15.6 (5.2)	7.1 (3.3)	46.3 (13.4)	
Gastroenterology	4.1 (2.4)	16.5 (6.3)	16.3 (6)	6.9 (3.2)	43.8 (13.4)	
Obstetrics and Gynecology	4.5 (2.1)	14.8 (6.7)	13.7 (6.7)	5.6 (3.9)	38.5 (15.1)	
Geriatrics	5.8 (3.4)	17.7 (6.6)	16.6 (6.6)	7.5 (3.8)	47.6 (16.4)	
Infectious diseases	5.3 (2.4)	15.8 (8.8)	14.1 (7.9)	7.1 (3.7)	42.2 (20.3)	
Emergency Medicine	6.1 (2.2)	17.6 (6.7)	14.2 (6.6)	6.8 (3.6)	44.7 (15)	
Physical medicine and Rehabilitation	4.1 (2)	13.4 (7.4)	12.3 (6.8)	5.4 (3.3)	35 (15)	
Intensivists and Critical Care	6.1 (2.5)	17.2 (6.3)	15 (5.7)	6.4 (3.6)	44.6 (14.6)	
Internal Medicine	5.8 (2.2)	16.6 (6.5)	15 (6.6)	6.1 (3.9)	43.5 (15.2)	
Nephrology	4.6 (2.2)	14 (6.6)	15.3 (5.1)	5.1 (3.1)	39 (15.1)	
Pneumology	6 (2)	18.7 (6.1)	15.8 (6.4)	7.8 (4.1)	48.3 (13.6)	
Neurosurgery	5.6 (2.1)	17.6 (6.1)	13.1 (5.8)	6.5 (3.7)	42.8 (13.5)	
Others	5.1 (2.7)	16.3 (7.2)	14.2 (7.2)	6.5 (4)	42.1 (17)	
Pediatrics	4.8 (2)	15.6 (6.5)	13.3 (6.2)	6.1 (3.6)	39.8 (15.2)	
Psychiatry	4.6 (2.4)	14.1 (7.6)	12.1 (6.7)	4.6 (3.7)	35.4 (17.1)	
Ambulance physicians	6.5 (2.7)	17.5 (5.9)	14.4 (6.6)	6.4 (3.8)	44.9 (14.9)	
Workplace						0.013
Primary hospital	5.3 (2.4)	15.8 (6.7)	13.8 (6.4)	6.2 (3.7)	41 (15.2)	
Secondary hospital	5.4 (2.6)	15.9 (6.6)	15 (6.9)	5.9 (3.6)	42.2 (15.9)	
Tertiary hospital	6 (2.6)	16.3 (6.5)	15.6 (7)	6.1 (3.8)	43.9 (16.6)	
General practitioners in medical centers	5.3 (2.4)	15.9 (6.7)	14.7 (6.6)	6.1 (3.8)	42.1 (15.6)	
Ambulance services	5.5 (2.7)	16.5 (6.5)	15.4 (6.4)	6.3 (3.8)	43.7 (15)	

*Group I: 19.7–33 cases per 100 000 people.

Group II: 34–70.8 cases per 100.000 people.

Group III: 70.9–117.9 cases per 100 000 people.

Group IV: 118–245.8 cases per 100 000 people.

Group V: 245.9–351.3 cases per 100 000 people.

** *p* values correspond to one-way ANOVA comparing the mean of *PSAS* by each of the categorical variables.

Tertiary hospital workers showed a higher level of stress, *PSAS* 43.9 (16.6) along with ambulance services, *PSAS* 43.7 (15) when compared to other groups (*p* < 0.0001). Seniority was a protective factor, PSAS 39.1 (15.2) (*p* < 0.0001). Other elements analyzed that might interfere in the psychological impact experimented are shown in Table 3. Respondents who felt they needed psychological support but did not have the time to receive it, showed a higher degree of stress, *PSAS* 52.5 (13.6) compared to those who did not need it, *PSAS* 39.7 (14.9) (*p* < 0.0001). Asymptomatic workers were less stressed with a *PSAS* 41.3 (15.4), than the symptomatic group, in isolation, or those who were positive in a COVID-19 test or were hospitalized (*p* < 0.001). Familiar exposure is also a determinant factor (*p* < 0.0001). Figure 2 shows a sub-analysis among different healthcare careers and work environment.

**Fig. 2.**
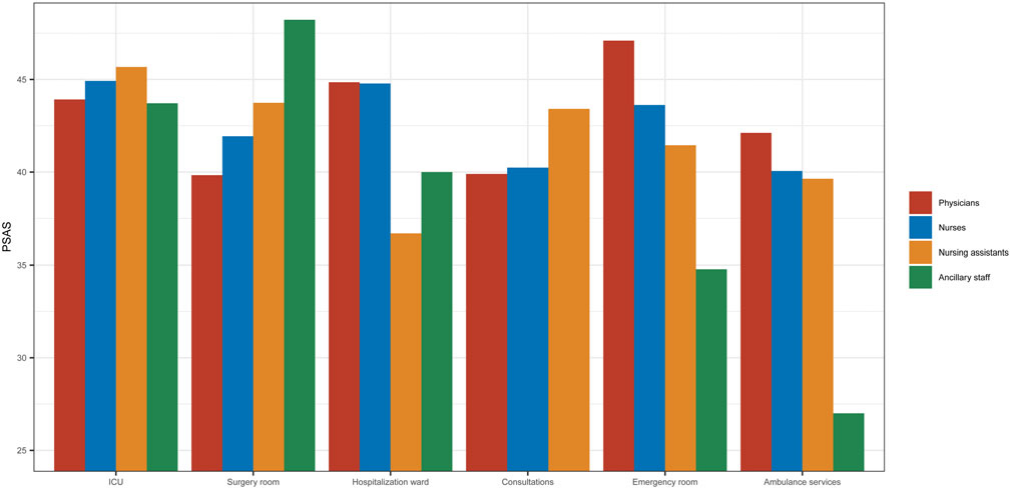
PSAS career mean by work environment.

**Table 3.** Precipitating factors and PSAS

Characteristics	No.	%	Total *n* = 3109	PSAS Mean (s.d.)	*p* value*
Children <12 years, elderly or handicapped at home					0.684
No	1640	53.4	1640 (53.4%)	42.2 (15.3)	
Yes	1429	46.6	1429 (46.6%)	42 (15.7)	
Living with your partner					0.096
No	742	23.9	742 (23.9%)	43.1 (15.8)	
Yes, not a healthcare worker	1538	49.5	1538 (49.5%)	41.8 (15.1)	
Yes, a healthcare worker	829	26.7	829 (26.7%)	41.8 (15.8)	
Work environment					0.012
ICU	605	19.5	605 (19.5%)	44.3 (15.4)	
Surgery room	599	19.3	599 (19.3%)	40.4 (15.3)	
Hospitalization ward	515	16.6	515 (16.6%)	43.3 (15)	
Consultations	354	11.4	354 (11.4%)	39.8 (15.6)	
Emergency department	316	10.2	316 (10.2%)	45.1 (16)	
Other	720	23.2	720 (23.2%)	40 (15.2)	
Psychotherapy					<0.001
No	2437	78.6	2437 (78.6%)	39.7 (14.9)	
No, but I would like to begin	453	14.6	453 (14.6%)	52.5 (13.6)	
Yes, I'm in therapy before the crisis	135	4.4	135 (4.4%)	49.2 (15.7)	
Yes, I've started therapy since the crisis	2	0.1	2 (0.1%)	55.5 (3.5)	
Other, non-conventional therapies	74	2.4	74 (2.4%)	45.4 (13.8)	
Personal exposure					<0.001
Asymptomatic	1953	63	1953 (63%)	41.3 (15.4)	
Symptomatic	704	22.7	704 (22.7%)	43.2 (15.5)	
In isolation	344	11.1	344 (11.1%)	44.3 (15.1)	
Positive in a test	91	2.9	91 (2.9%)	43.7 (16.1)	
I've been hospitalized in a ward	7	0.2	7 (0.2%)	45.9 (10)	
I've been hospitalized in the ICU	0	0	0 (0%)		
Family exposure					<0.001
No	2376	76.7	2376 (76.7%)	41.5 (15.5)	
Yes	721	23.3	721 (23.3%)	44.2 (15.4)	

*p values correspond to one-way ANOVA comparing the mean of *PSAS* by each of the categorical variables.

The psychological impact of the COVID-19 pandemic in healthcare workers in Spain, has been evaluated. The stress level perceived is predominant in workers that have been in contact directly with COVID-19 patients, like Respiratory Medicine, and in those with family exposure. In the Emergency Medicine (Portero de la Cruz, Cebrino, Herruzo, & Vaquero-Abellán, 2020), workers have also suffered a high impact. This may be indicative that in this environment, COVID-19 exposure is uncertain. The protective effect of seniority may be due to the fact that, expertise and confidence, helps minimizing the stress caused by unforeseen situations. The number of cases in a geographical area was also a conditioning element for the stress. The higher the incidence the disease is, the more stressed the healthcare workers feel (Xiao et al., 2020).

This study has several limitations, the critical nature of the emergency, did not allow to obtain a previous assessment of stress levels or the use of an extended version of the tests. More than 66% of the respondents were working on the second least-affected area, so the reported stress impact could be underestimated.

To the best of our knowledge, this is the largest psychological impact study on healthcare workers during a major pandemic crisis, to date(Kang et al., 2020). Psychological support has demonstrated to minimize the negative impact on healthcare workers. Novel therapy approaches such as on-line support, mindfulness, relaxation therapies, etc. may have a promising role (Xiao, 2020; Yang, Yin, Duolao, Rahman, & Xiaomei, 2020) when the lack of time is a precipitating agent. A second survey will be carry out to assess stress levels among healthcare workers after the crisis finally ends.

## Supplementary material

For supplementary material accompanying this paper visit http://dx.doi.org/10.1017/S0033291720001671. click here to view supplementary material

## *PSIMCOV NETWORK

Ricardo Salcedo, MD, Medical Director, Hospital General de Valencia, Valencia (Spain); Pilar Albors, MD, Hospital General de Valencia, Valencia (Spain); Pablo Alcocer, MD, Hospital Nisa 9 de Octubre, Valencia (Spain); Mónica Álvarez, Hospital Universitario de Burgos, Burgos; Mara Andrés, MD, Hospital La Fe, Valencia (Spain); Fernando Antón, Anaford Abogados, Valencia (Spain); Francisco de Asís Aparisi, MD, Hospital General de Valencia, Valencia (Spain); Daniel Arnal, MD, Hospital de Alcorcón, Madrid (Spain); Carmen Baixauli, MD, Hospital General de Valencia, Valencia (Spain); Maria Teresa Ballester, MD, Hospital General de Valencia, Valencia (Spain); Laura Barragán, MD, Centro de salud el Vedat de Torrente, Valencia (Spain); Vibiana Blanco, MD, Centro de salud Trafalgar, Valencia (Spain); Paula Bovaira, Hospital Intermutual, Valencia (Spain); Maria Brugada, MD, Hospital La Fe, Valencia (Spain); Nerea Bueno, MD, SAMU, Valencia (Spain); Antonio Cano, Hospital General de Valencia, Valencia (Spain); Eva Carbajo, Hospital General de Valencia, Valencia (Spain); Beatriz Carrasco, MD, Hospital Virgen de la Luz, Cuenca (Spain); Marta Castell, MD, Hospital La Fe, Valencia (Spain); Isabel Catalá, diseño gráfico, Valencia (Spain); Pablo Catalán, MD; Hospital Arnau de Vilanova, Valencia (Spain); Lucía Cervera, Southport District General Hospital, Liverpool (UK); Irina Cobo, MD, Hospital General de Valencia, Valencia (Spain); Dolores de las Marinas, MD, Hospital General de Valencia, Valencia (Spain); Gema del Castillo, MD, Hospital de Sagunto, Valencia (Spain); Alejandro Duca, MD, Hospital de Manises, Valencia (Spain); Juana María Elía JM, MD, Hospital General de Valencia, Valencia (Spain); Cristina Esteve, Hospital General de Valencia, Valencia (Spain); Silvia Ferri, MD, Hospital de Torrejón, Madrid (Spain); Juana Forner, MD, Hospital General de Valencia, Valencia (Spain); Óscar Gil, MD, Hospital General de Valencia, Valencia (Spain); Marta Gómez-Escolar, MD, Emergencias Sanitarias Castilla y León, Valladolid (Spain); Rosa Hernández, MD, Hospital General de Valencia, Valencia (Spain); Vega Iranzo, MD, Hospital General de Valencia, Valencia (Spain); Raúl Incertis, MD, Hopital de Manises, Valencia (Spain); Ana Izquierdo, MD, Hopital de Manises, Valencia (Spain); María Teresa Jareño, Hospital General de Valencia, Valencia (Spain); Eva Jordá, Hospital General de Valencia, Valencia (Spain); Nela Klein-González, MD, Hospital Clínic de Barcelona, Barcelona (Spain); Pau Klein-González, digital marketing, Valencia (Spain); Amparo Lluch, MD, Hospital General de Valencia, Valencia (Spain); María Dolores López, MD, Hospital General de Valencia, Valencia (Spain); Sara López, MD, Hospital General de Valencia, Valencia (Spain); Eva Mateo, MD, Hospital General de Valencia, Valencia (Spain); Amanda Miñana, MD, Hospital de Gandía, Valencia (Spain); Sergio Mont, Hospital General de Valencia, Valencia (Spain); Maria Carmen Navarro, Researcher, Fundación Hospital General de Valencia (Spain); Pilar Ortega, Hospital General de Valencia, Valencia (Spain); Alessandro Pirola, MD, Hospital General de Valencia, Valencia (Spain); Pablo Renovell, MD, Hospital General de Valencia, Valencia (Spain); Esther Romero, MD, Hospital Clínico de Valencia, Valencia (Spain); Javier Ripollés, Hospital Infanta Leonor, Madrid (Spain); Susana Royo, MD, Hospital General de Valencia, Valencia (Spain); Juan Ramón Ruiz, MD, Hospital General de Valencia, Valencia (Spain); Moisés Sánchez, MD, Hospital General de Valencia, Valencia (Spain); Nerea Sanchís, MD, Hospital General de Valencia (Spain); Francisco Sanz, MD, Hospital General de Valencia, Valencia (Spain); Silvana Serrano, Hospital General de Valencia, Valencia (Spain); Jose Luis Soriano, MD, NHS Bedfordshire Hospitals, Bedford (UK); Ana Tirado, MD, Hospital Infanta Leonor, Madrid (Spain); Francisco Verdú, MD, Hospital General de Valencia, Valencia (Spain); Enrique Zapater, MD, Hospital General de Valencia, Valencia (Spain). In collaboration with SEDAR (Sociedad Española de Anestesia, Reanimación y Terapeútica del dolor), SEPYPNA (Sociedad Española de Psiquiatría y Psicoterapia del Niño y el Adolescente), SENSAR, REDGERM y el COMV (Colegio Oficial de Médicos de Valencia).

## References

[ref1] Aranaz, J., Mira, J., & Font-Roja Questionnaire. (1988). A measurement tool for satisfaction in hospital environment (in Spanish). Todo Hospitales, 52, 63–66.

[ref2] Cano, F. J., Rodríguez, L., & García, J. (2007). Spanish adaptation of coping strategies inventory. Actas Españolas de Psiquiatría, 35(1), 29–39. 17323223

[ref3] Carver, C. S., Scheier, M. F., & Weintraub, J. K. (1989). Assessing coping strategies: A theoretically based approach. Journal of Personality and Social Psychology, 56, 267–283. 2926629 10.1037//0022-3514.56.2.267

[ref4] Casas, J., Repullo, J. R., & Lorenzo, S. (2002). Stress at work in healthcare environment and adaptative coping strategies (in Spanish). Revista de Calidad Asistencial, 17(4), 237–246.

[ref5] Core Team (2013). R: A language and environment for statistical computing. Vienna, Austria: R Foundation for Statistical Computing Retrieved from http://www.R-project.org/.

[ref6] Fernández-Berrocal, P., & Extremera, N. (2006). Emotional intelligence investigation in Spain (in Spanish). Ansiedad y Estrés, 12(2–3), 139–153.

[ref7] Haynes, S., & Lench, H. (2003). Incremental validity of new clinical assessment measures. Psychological Assessment, 15(4), 456–456. 14692842 10.1037/1040-3590.15.4.456

[ref8] Johnston, C., & Murray, C. (2003). Incremental validity in the psychological assessment of children and adolescents. Psychological Assessment, 5(4), 496–507. 10.1037/1040-3590.15.4.496 14692845

[ref9] Kang, L., Ma, S., Chen, M., Yang, J., Wang, Y., Li, R., & Liu, Z. (2020). Impact on mental health and perception of psychological care among medical and nursing staff in Wuhan during the 2019 novel coronavirus disease outbreak: A cross-sectional study. Brain, Behavior and Immunity, S0889–1591(20), 30348–2. doi: 10.1016/j.bbi.2020.03.028. PMC7118532 32240764

[ref10] Pfefferbaum, B., & North, C.S. (2020). Mental health and the Covid-19 pandemic. New England Journal of Medicine doi:10.1056/NEJMp2003149. 32283003

[ref11] Portero de la Cruz, S., Cebrino, J., Herruzo, J., & Vaquero-Abellán, M. (2020). A multicenter study into burnout, perceived stress, job satisfaction, coping strategies, and general health among emergency department nursing staff. Journal of Clinical Medicine, 9(4), E1007. 32252444 10.3390/jcm9041007 PMC7230883

[ref12] Salovey, P., Mayer, J., Goldman, S., Turvey, C., & Palfai, T. (1995). Emotional attention, clarity, and repair: Exploring emotional intelligence using the trait meta-mood scale In Pennebaker J. (Ed.), APA Science volume series (pp. 125–154). Washington, DC: American Psychological Association.

[ref13] Tobin, D. L., Holroyd, K. A., Reynolds, R. V., & Kigal, J. K. (1989). The hierarchical factor structure of the coping strategies inventory. Cognitive Therapy Research, 13(4), 343–361.

[ref14] Xiao, C. (2020). A novel approach of consultation on 2019 novel coronavirus (COVID-19)-related psychological and mental problems: Structured letter therapy. Psychiatry Investigation, 17(2), 175–176. 32093461 10.30773/pi.2020.0047 PMC7047000

[ref15] Xiao, H., Zhang, Y., Kong, D., Li, S., & Yang, N. (2020). The effects of social support on sleep quality of medical staff treating patients with coronavirus disease 2019 (COVID-19) in January and February 2020 in China. Medical Science Monitor, 26, e923549. 32132521 10.12659/MSM.923549 PMC7075079

[ref16] Yang, L., Yin, J., Duolao, W., Rahman, A., & Xiaomei, L. (2020). Urgent need to develop evidence-based self-help interventions for healthcare workers in COVID-19 pandemic. Psychological Medicine, 28, 1–3. 10.1017/S0033291720001385 PMC7203165 32340642

[ref17] Yuan, S., Liao, Z., Huang, H., Jiang, B., Zhang, X., Wang, Y., & Zhao, M. (2020). Comparison of the indicators of psychological stress in the population of Hubei province and non-endemic provinces in China during two weeks during the coronavirus disease 2019 (COVID-19) outbreak in February 2020. Medical Science Monitor, 26, e923767. 32294078 10.12659/MSM.923767 PMC7177041

