# Supplementary material for: COVID-19 psychological impact in 3109 healthcare workers in Spain: The PSIMCOV group
Source: Psychol Med. 2020 May 14:1–7. doi: 10.1017/S0033291720001671 (PMC7477466; doi:10.1017/S0033291720001671)
Supplement: Supplementary file 1 [file S0033291720001671sup001.docx]

Appendix 1 – Stress and adaptation at work survey for healthcare professionals during COVID-19 pandemic emergency.

- **General questions:**

1. Age: ______
2. Geographical area where you work: ________________________________________
3. State your professional career (physician, nurse, assistant, manager, technician…): ______________________________________________________________________
4. What is your medical specialty? ____________________________________________
5. I am currently working at a:

- Primary Care Center.
- First-level hospital.
- Second-level hospital.
- Third-level hospital.

1. I had contact with COVID-19 positive patients:

- Yes.
- No.

1. My type of working contract is:

- Permanent.
- Temporary.
- I was hired specifically for this emergency.

1. My job experience is:
   - 0-1 year.
   - 1-3 years.
   - 3-5 years.
   - 5-10 years.
   - 10-20 years.
   - >20 years.
2. Do you have any dependents (children under 12 years old, disabled…)?
   - Yes.
   - No.
3. Are you living with a partner at the moment?
   - No.
   - Yes, and he/she belongs to the healthcare environment.
   - Yes, and he/she does not belong to the healthcare environment.
4. In which area are you working at the moment?
   - Intensive Care.
   - Surgical Area.
   - Hospitalization Ward.
   - Outpatient Visits.
   - Emergency Room.
   - Other.
5. I have personally been affected by coronavirus to the following extent:
   - I have been asymptomatic.
   - I have had symptoms.
   - I have been quarantined.
   - I tested positive for COVID-19.
   - I was admitted in the Hospitalization Ward.
   - I was admitted in the ICU.

- **Questionnaire A: Modified Healthcare Stressful Factors Test: State how frequently the following situations have been stressful to you since the present emergency started.**

1. I felt hopeless regarding to a patient not responding to treatment or suffering.

Never Sometimes Often Always

1. I felt alone when a patient was in an emergency situation or dying.

Never Sometimes Often Always

1. I did not know how to manage new or specialized equipment.

Never Sometimes Often Always

1. I received negative comments from my colleagues.

Never Sometimes Often Always

1. I have been in disagreement with task or resource management at work.

Never Sometimes Often Always

- **Questionnaire B: Modified Coping Strategies Inventory: Think about a stressful situation you have experienced at work since the beginning of the current pandemic. State what you have experimented of the following situations explained. Please, rate yourself honestly based on what you actually feel following the scale:**

**6 Always 5 Very often 4 Often 3 Sometimes 2 Rarely 1 Never**

1. I tried to avoid facing a problem

1 2 3 4 5 6

1. I blamed myself for something that happened.

1 2 3 4 5 6

1. I avoided to express my feelings with my peers during or after the emergency.

1 2 3 4 5 6

1. I wished a situation not to be happening or not to have happened at all.

1 2 3 4 5 6

1. I had a bad reaction in the presence of other people to express contained emotions.

1 2 3 4 5 6

1. I chose not to analyze the causes of something that happened and avoided introspection so that I could keep doing my tasks.

1 2 3 4 5 6

1. I took action before thinking because I was feeling overwhelmed.

1 2 3 4 5 6

1. I avoided spending time with people from my environment and I isolated myself because of my personal distress.

1 2 3 4 5 6

- **Questionnaire C: Modified Font-Roja Satisfaction at Work Test: Choose to what extent the following situations described above match the way you currently perceive your work**. **Please, rate yourself honestly based on what you actually feel following the scale:**

**6 Always 5 Very often 4 Often 3 Sometimes 2 Rarely 1 Never**

1. I feel unsatisfied at work and poorly recognized.

1 2 3 4 5 6

1. I feel my workload is overwhelming and I don’t have time to complete my tasks.

1 2 3 4 5 6

1. Social relationships feel unfulfilling to me.

1 2 3 4 5 6

1. I feel poorly remunerated for my job.

1 2 3 4 5 6

1. I don’t feel properly trained for my job.

1 2 3 4 5 6

1. I feel unable to organize my time or to learn new things at work.

1 2 3 4 5 6

1. I feel my job is monotonous and repetitious.

1 2 3 4 5 6

- **Questionnaire D: Modified Trait Meta-Mood Scale Test: Read the following sentences about your feelings and emotions since COVID-19 pandemics started. Please, rate yourself honestly based on what you actually feel following the scale:**

**6 Always 5 Very often 4 Often 3 Sometimes 2 Rarely 1 Never**

1. I believe I don’t think about my feelings very much.

1 2 3 4 5 6

1. I usually don’t fully understand why I feel sad or why I think too much about things.

1 2 3 4 5 6

1. Even though I know I must focus on the positive side of things, I find it very hard to do.

1 2 3 4 5 6

- **Miscellaneous items related to physical and psychological well-being during COVID-19 pandemic, not included in *PSAS* score. Please, rate yourself honestly based on what you actually feel following the scale:**

**6 Always 5 Very often 4 Often 3 Sometimes 2 Rarely 1 Never**

1. I feel physically overloaded at work.

1 2 3 4 5 6

1. I feel emotionally overloaded at work.

1 2 3 4 5 6

1. I feel my health is threatened because of actual risks at work.

1 2 3 4 5 6

1. I feel disappointed with the work dynamics established at my job.

1 2 3 4 5 6

1. I feel unsatisfied with the hierarchical structure stablished at work.

1 2 3 4 5 6

1. The fact of finding myself within an unfamiliar environment and team affects my personal stability and performance at work.

1 2 3 4 5 6

1. I feel my job is not making any difference to social advancements towards resolution.

1 2 3 4 5 6

1. I am afraid of whatever may happen after this health emergency. State your level of agreement.

1 2 3 4 5 6

1. I feel I can’t balance my family life because of my job. State your level of agreement.

1 2 3 4 5 6

1. I am receiving some kind of psychological support at the moment

No

No, but I would like to.

Yes, I was in therapy before the emergency.

Yes, I am in therapy since the emergency began.

I am getting other forms of psychological support rather than conventional psychotherapy.
